# Antioxidant Properties of Fruit and Vegetable Whey Beverages and Fruit and Vegetable Mousses

**DOI:** 10.3390/molecules26113126

**Published:** 2021-05-24

**Authors:** Aleksandra Purkiewicz, Renata Pietrzak-Fiećko

**Affiliations:** Department of Commodities and Food Analysis, Faculty of Food Sciences, University of Warmia and Mazury in Olsztyn, 1 Cieszyński Square, 10-726 Olsztyn, Poland; aleksandra.purkiewicz@uwm.edu.pl

**Keywords:** whey beverages, mousses, fruits, vegetable, antioxidant activity, antioxidants

## Abstract

The study assesses the antioxidant activity, total phenolic content, total flavonoids content and lipophilic pigments (β-carotene, chlorophyll a, chlorophyll b) content in homemade and marketed fruit and vegetable whey beverages and fruit and vegetable mousses. All of the tests were performed using spectrophotometric methods. The highest polyphenol content was found in the homemade green whey beverage W1G (541.95 mg/100 g) and the lowest in the market green whey beverage W2G (46.18 mg/100 g). In the fruit and vegetable mousses under study, the highest content of polyphenolic compounds was determined in the red mousse R3 (76.41 mg/100 g). The highest content of flavonoids was observed in the homemade orange whey beverage W1O (63.06 mg/100 g) and in the green mousse G2 (69.80 mg/100 g). The values of the antioxidant activity of whey beverages and mousses varied depending on the composition. The highest content of β-carotene was identified in homemade orange whey beverage (4.36 mg/100 g) and in orange mousses (in range 1.10–2.24 mg/100 g), while chlorophylls a and b—in homemade green whey beverage W1G (3.00 mg/100 g and 1.31 mg/100 g respectively) and in green mousses (chlorophyll a in range 0.54 to 1.42 mg/100 g and chlorophyll b in range 0.13 to 0.32 mg/100 g).

## 1. Introduction

Fresh and processed fruits and vegetables are known for their significant antioxidant activity due to the high content of various antioxidants. The antioxidant activity is an excellent example of the functional benefits of consuming plant-based foods. The content of selected bioactive compounds in fruit and vegetables depends on the type of raw material. Carotenoids are found mainly in yellow, orange and red fruits and vegetables. Large amounts of α- and β-carotene are found in carrots, pumpkins, mangoes and peaches; the greatest amounts of lycopene are accumulated in tomatoes, while green leafy vegetables are a good source of lutein and zeaxanthin [1]. Polyphenolic compounds, which include flavonoids, phenolic acids, lignans and stilbenes, are found in products such as fruits, vegetables, coffee, tea, herbs and spices. The flavonoids include, among others flavonols (kaempferol, quercetin, myricetin), flavanols (catechin, epicatechin), found in cocoa, dark chocolate or green and black tea, and anthocyanins, the source of which are berries [2]. Compounds with strong antioxidant properties also include betalains, found in red beet, cactus pear, pitaya and amaranth [3] and chlorophylls found in leafy green vegetables [1].

Beverages are a product in great demand on the market due to their sensory properties and health-promoting value. They constitute an important part of the total diet among a large percentage of the population [4]. The beverage and beverage market are constantly evolving and producers are trying to meet the growing needs of consumers. The production of beverages is becoming an interesting and very promising direction for the use of whey. Whey is a by-product of cheese production. More often, this product is utilized as an additive to various types of food products, but it is still often considered a post-production waste. Depending on the type of casein coagulation, there are two types of whey: sweet and acid. The food industry constantly creates new products with increased nutritional value compared to traditional ones, including products enriched with whey [5]. Whey can be a water substitute in the preparation of beverages with the addition of fruit and vegetables [5]. Fruit and vegetable beverages based on whey are considered to be an innovative functional product taking into account the technological process [6]. Producing whey-based beverages can be a good method of increasing the health benefits of beverages due to the fact that acid whey is rich in valuable proteins with high nutritional value; caseinomacropeptide, which protects against bacteria and viruses and stimulates the immune system; lactose which has prebiotic properties and enhances the absorption of calcium and magnesium and minerals, which regulate physiological functions, including proper bone development [6,7], and fruit and vegetables are a valuable source of antioxidants [8]. The combination of these two components for the production of beverages gives the possibility to create products with increased health-promoting properties.

Numerous scientific reports confirm the high antioxidant activity of fruit and vegetable beverages and mousses. The health-promoting properties of fruit and vegetables are determined primarily by the content of bioactive compounds such as polyphenolic compounds, including flavonoids, carotenoids, betalains or vitamin C [8]. So far, no attempt has been made to determine the antioxidant properties of fruit and vegetable drinks based on whey. The innovative element of the research results from the determine of the influence of type and amounts fruit and vegetable components on the anti-oxidant activity and amounts of selected antioxidants in whey beverages. Furthermore, innovative element of the research was to assess the antioxidant properties of not studied before homemade and market fruit and vegetable whey beverages. Moreover, such a wide assortment of fruit and vegetable mousses has not been previously studied. The aim of the study was to determine and compare the antioxidant properties of homemade and commercial fruit and vegetable whey beverages and fruit and vegetable mousses.

## 2. Results

### 2.1. Antioxidant Properties of Homemade and Market Fruit and Vegetable Whey Beverages

#### 2.1.1. Total Phenolic Content (TPC) and Total Flavonoids Content (TFC)

The results of the total phenolic compounds and flavonoids content in the analyzed homemade and market fruit and vegetable whey beverages are presented in Table 1 Among the whey beverages, the highest polyphenol content was found in the homemade green whey beverage (W1G) (*p* < 0.05) and the lowest in the market green whey beverage (W2G). The polyphenol content in the homemade whey beverages was respectively over eight times, three and a half times and twelve times higher than in the market whey beverages in these colors. The content of phenolic compounds did not differ significantly in the case of the market orange (W2O) and green (W2G) whey beverage.

The highest content of flavonoids was observed in the homemade orange whey beverage (W1O) (*p* < 0.05). It was more than one and a half times higher than in the homemade red (W1R) and green (W1G) whey beverages and about four and a half times higher than in the market whey beverages (W2O, W2R, W2G). The content of flavonoids did not differ significantly in the homemade red (W1R) and green (W1G) whey beverages and the market orange (W2O), red (W2R) and green (W2G) whey beverages. The lowest content of flavonoids was identified in market orange (W2O) and red (W2R) whey beverages (*p* < 0.05).

#### 2.1.2. Antioxidant Activity

Two tests (DPPH, ORAC) were used to determine the antioxidant activity in the analyzed homemade and market fruit and vegetable whey beverages. The results of the antioxidant activity are presented in Table 1. The antioxidant activity measured by the DPPH test was at the highest level in the homemade green whey beverage (W1G). This activity did not differ significantly from that of the homemade orange (W1O) and red (W1R) whey beverages. The antioxidant activity of the homemade whey beverages was seven, eleven and six times lower in the market orange, red and green whey beverages. The antioxidant activity of market orange (W2O) and green (W2G) whey beverages did not differ statistically. Market red whey beverage (W2R) had the lowest antioxidant activity.

The antioxidant activity measured by the ORAC test was the highest in homemade red whey beverage (W1R). The values of the antioxidant activity of homemade orange, red and green whey beverages did not differ significantly (*p* < 0.05). Homemade orange, red and green whey beverages had respectively 39%, 38% and 13% higher levels of antioxidant activity than market whey beverages in these colors. Market orange (W2O) and red (W2R) whey beverages had the lowest values of antioxidant activity.

The results of the antioxidant activity in the homemade orange, red and green whey beverages measured with the DPPH test were higher than in the ORAC test by 10%, 6% and 12%, respectively. The opposite tendency was observed in the case of market whey beverages—higher values of antioxidant activity (five, seven and almost five times, respectively) were determined in the ORAC test.

#### 2.1.3. Lipophilic Pigments

The results of the content of lipophilic pigments in the analyzed homemade and market fruit and vegetables whey beverages are presented in Table 1. Among the lipophilic pigments found in the analyzed homemade and marketed fruit and vegetable whey beverages, compounds belonging to the group of carotenoids (β-carotene) and chlorophylls (chlorophyll a and chlorophyll b). The highest content of β-carotene was determined in the homemade orange whey beverage (W1O) (*p* < 0.05). Its content was over five, twenty and two hundred and eighteen times lower in the homemade green whey beverage (W1G) and market orange (W2O) and green (W2G) whey beverages. The higher level of chlorophyll a was in the homemade green whey beverage (W1G). The homemade and market red whey beverages contained over 33 times lower amounts of this compound, and the homemade orange whey beverage and market green and orange whey beverages contained 150, 150 and 300 times less, respectively. A similar tendency was observed in the content of chlorophyll b—the highest content was observed in the homemade green whey beverage. The content of chlorophyll b was ten times lower in the homemade and market whey beverages and several dozen times lower in the market green, homemade orange and market orange whey beverages compared to the homemade green whey beverage.

### 2.2. Antioxidant Properties of Fruit and Vegetable Mousses

#### 2.2.1. Total Phenolic Content (TPC) and Total Flavonoids Content (TFC)

The results of the total phenolic compounds and flavonoids content in the analyzed fruit and vegetable mousses are presented in Table 1.

In the fruit and vegetable mousses under study, the highest content of polyphenolic compounds was determined in the red mousse R3. It was by 44%, 36% and 36% higher than in orange mousses O1, O2 and O3; 34% and 7% higher than in red mousses R1 and R2 and 26%, 9% and 19% higher than in green mousses G1, G2 and G3. The content of phenolic compounds did not differ significantly in red mousse R2 and green G2 and G3 (*p* < 0.05) The lowest content of phenolic compounds was found in orange mousse O1. Similar values of phenolic compounds were observed in orange mousses O1, O2 and O3, red R1 and green G1.

The content of flavonoids was at the highest level in the green mousse G2. This content did not differ significantly from this in the green mousses G1 and G3 and orange mousse O3 (*p* < 0.05), but it differed from the content of flavonoids in the other mousses. The amount of flavonoids in green mousse G2 was 27%, 52% and 9% higher than in orange mousses O1, O2 and O3; 62%, 29 and 85% higher than in red mousses R1, R2 and R3. The content of flavonoids did not differ significantly in orange mousse O1 and red mousse R2 and in orange mousse O2 and red R1. The lowest total flavonoid content was determined in red mousse R3.

#### 2.2.2. Antioxidant Activity

Two tests (DPPH, ORAC) were used to determine the antioxidant activity in the analyzed fruit and vegetable mousses. The results of the antioxidant activity are shown in Table 1.

The significantly highest antioxidant activity measured with the DPPH test was determined in the red mousse R2 (*p* < 0.05). It was by 39%, 55% and 112% higher than in orange mousses O1, O2 and O3; 59% and 8% higher than in red mousses R1 and R3 and by 118%, 17% and 128% higher than in green mousses G1, G2 and G3. There were no statistical differences in the amount of antioxidant activity in red R3 and green G2 and in orange O1, O2 and red R1 mousses. Green mousse G3 had the lowest antioxidant activity. The value of its antioxidant activity was similar to the activity value of orange mousse O3 and green mousse G1.

O3 orange mousse was characterized by the highest antioxidant activity as measured by the ORAC test. This activity was higher by over 20% than in the orange mousses O1 and O2; 16%, 77% and 21% higher than in R1, R2 and R3 red mousses and 36%, 24% and 164% higher than in G1, G2 and G3 green mousses. The antioxidant activity of O1, O2, R1, R3 and G2 mousses did not differ significantly.

#### 2.2.3. Lipophilic Pigments

The results of the content of lipophilic pigments in the analyzed fruit and vegetables mousses are presented in Table 1. The content of individual lipophilic pigments in the analyzed fruit and vegetable mousses varied depending on the raw material composition. The orange mousses contained the most β-carotene. O2 mousse was determined with the highest amounts of β-carotene—about twice as much as O1 and O3 mousses. Among green mousses, the greatest amount of β-carotene was found in G1 mousse, which contained almost twice as much of this compound as G3 mousse and over 3 times more than G2 mousse. Red mousses were the most deficient in β-carotene. The β-carotene content in the R1 and R2 mousse was 28 times lower than in the O2 mousse.

The highest levels of chlorophyll a were accumulated by green mousses (0.54–1.42 mg/100 g). G1 mousse contained significantly the highest amount of this compound, by 29% and 162% more than G3 and G2. Chlorophyll b content was also dominant in green mousses (0.13–0.32 mg/100 g). The relatively high content of chlorophyll b was found in O2 mousse (0.11 ± 0.06 mg/100 g) and R1 (0.10 ± 0.01 mg/100 g). The lowest level of chlorophyll b was identified in the mousse O1, O3 and R2.

### 2.3. Association Between Obtained Data

#### 2.3.1. Linear Pearson’s Correlation Coefficients

The relationship between the tests of antioxidant activity and the compounds of antioxidant nature is presented in Table 2. There was a weak positive correlation between DPPH and ORAC antioxidant activity tests (r = 0.43). A strong positive relationship was observed between the DPPH test and TPC (r = 0.77), DPPH and TFC (r = 0.61), ORAC test and TPC (r = 0.68), significant at the significance level of *p* < 0.05. There was a strong positive correlation between total chlorophylls content and TPC (r = 0.61), and positive correlation between total chlorophylls content and DPPH test (r = 0.32) and ORAC test (r = 0.14). Moreover, a significant linear relationship was observed between β-carotene and TFC (r = 0.53).

#### 2.3.2. Principal Component Analysis (PCA)

**Figure 1:**
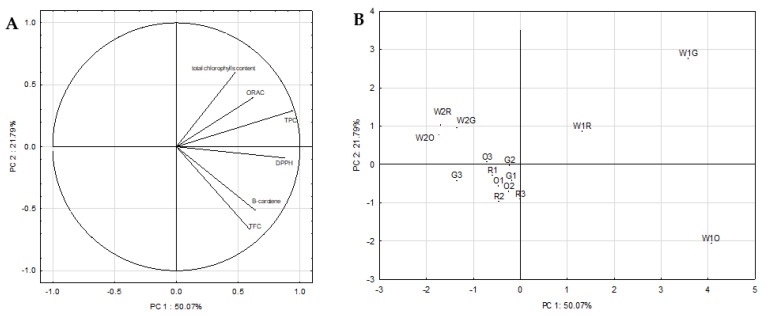
Principal components plot, variations in the parameters (TPC, TFC, DPPH, ORAC, total chlorophylls content and β-carotene) of the analyzed fruit and vegetable whey beverages and fruit and vegetable mousses (**A**) score plot of the analyzed fruit and vegetable whey beverages and fruit and vegetable mousses (**B**). Explanations: W1O, W1R, W1G—homemade fruit and vegetable whey beverages; W2O, W2R, W2G—market fruit and vegetable whey beverages; O1, O2, O3—orange mousses; R1, R2, R3—red mousses, G1, G2, G3—green mousses.

Principal component analysis (PCA) was used to explain the variability structure of the obtained data. The PCA was carried out among all studied samples and variables (total phenolic compounds content, total flavonoid content, antioxidant activity tested with DPPH and ORAC tests, content of lipophilic pigments—total chlorophylls, β-carotene). The two principal components describe 71.77% of the variance. Figure 1a shows the inter-correlations between the individual variables and the resulting principal components. The direction and lengths of the vectors indicate to what extent the given variables affect the principal components. In the research, some of the input variables (TPC, TFC, DPPH) are located near the circle, which means that the information contained in them is transferred by the principal components, while the information in the other input variables (ORAC, β-carotene, total chlorophylls content) is transferred to a smaller degree are transferred by the principal components. PCA showed a positive relationship between DPPH and TPC, DPPH and TFC, and ORAC and TPC. Moreover, a positive correlation was observed between total chlorophylls content and DPPH, ORAC, TPC and TFC, and a weak negative correlation between total chlorophylls content and β-carotene.

Figure 1b shows a plot of points in the plane of the principal components which illustrates the similarity between the antioxidant properties of the studied whey beverages and fruit and vegetable mousses. The arrangement of the analyzed cases in relation to each other proves the different antioxidant properties and the content of bioactive compounds in the studied homemade fruit and vegetable whey beverages. This is due to the content of various bioactive compounds included—the homemade orange whey beverage contained significant amounts of β-carotene, and homemade green whey beverage—chlorophylls. Based on the diagram, market fruit and vegetable whey beverages were similar in terms of antioxidant properties and bioactive compounds content, which is due to their similar composition. There was a similarity between the analyzed fruit and vegetable mousses—regardless of the color of the mousses (orange, red, green), the cases were concentrated in one point on the graph. It proves similar antioxidant abilities and a similar content of bioactive compounds (polyphenols, flavonoids, lipophilic pigments).

## 3. Discussion

The content of phenolic compounds and flavonoids, as well as the antioxidant activity of individual fruit and vegetable products, varied depending on the composition of the product.

The highest content of phenolic compounds in whey beverages was determined in the homemade green whey beverage (W1G). This results from the fact that parsley, constituting 25% of the beverage in question, is particularly rich in phenolic compounds (13,600 mg/100 g) [9]. The high content of phenolic compounds in whey-based beverages is also due to the fact that the Folin-Ciocalteu reagent, used for the color reaction during the determination of polyphenolic compounds, has the ability to react with amino acids, e.g., with tyrosine, tryptophan and cysteine [10]. Whey contains whey proteins [7] with which the Folin-Ciocalteu reagent could react, giving the overestimated results of polyphenolic compounds. Among the market whey beverages, the red beverage (W2R) was characterized by the highest amount of phenolic compounds. Market whey beverages were characterized by lower antioxidant parameters compared to homemade whey beverages, due to a much lower input of fruit and vegetables. In the analyzed beverages, fruit and vegetables are responsible for the increase in antioxidant activity. Their addition, except from enriching the sensory properties of whey, allowed to modulate the functional properties of the beverage and increase their nutritional value [6].

Rizollo and Cortellino [6] indicate that the content of polyphenolic compounds in “yellow” whey beverages based on apple was 48.7 ± 0.95 mg/100 g, and in the base of pear 34.6 ± 0.35 mg/100 g. The results obtained by the authors were the closest to those of the market orange (W2O) and green (W2G) whey beverages under study. On the other hand, it is difficult to compare the beverages of the above-mentioned authors to the analyzed beverages due to the different amounts of raw materials used to prepare the beverages. In the research by Rizollo and Cortellino [6], the share of fruit and vegetables was 80%, while the studied homemade whey beverages contained 50%, and market beverages from 6 to 8%. In the cited studies, the authors also undertook research on red beverages, based on blueberries and strawberries. The content of polyphenolic compounds in these beverages was respectively 310.7 ± 1.9 mg/100 g and 245.2 ± 1.8 mg/100 g. The content of phenolic compounds in analyzed red whey beverage was 25% lower than in the blueberry-based beverage tested by the authors and 1% higher than in a strawberry-based beverage. The different amounts of phenolic compounds differ mainly in the raw composition of the beverages. The differences in the content of phenolic compounds could also be influenced by the pasteurization of beverages used by the authors. Many studies show that the temperature used during pasteurization has a significant effect on reducing the content of polyphenols [11].

In the study by Sabokbar and Khodaiyan [12], the phenolic compounds content of the whey-based beverage with the addition of pomegranate was 10.1 mg/100 g. These values were several dozen times higher than in the homemade whey beverages under study (W1O, W1R, W1G) and several times higher than in commercial whey beverages (W2O, W2R, W2G). Different results indicate a different amount of plant raw materials added. The homemade whey beverages contained 50% of fruit and vegetables, while the market whey beverages—from 6.5 to 8%. In the studies of the cited authors, less plant material was used than in the authors’ own research, hence significantly lower values of antioxidant activity were obtained. Jaworska et al. studied the content of phenolic compounds in whey beverages enriched with 12.5% addition of blackcurrant beverage, which contained 111 mg of polyphenols in 100 g of the product. The value is almost four, two and five times lower than in homemade orange, red and green whey beverages, and twice, one and a half times and twice as high as in market orange, red and green whey beverages. The different content of phenolic compounds is dependent on the different share of plant material in the beverages. Studied homemade whey beverages contained four times higher, and market whey beverages by almost 5% lower addition of fruit and vegetables than in the studies by Jaworska et al. [13]. The authors also conducted research on whey beverages with the addition of apple beverage. The content of phenolic compounds in these beverages was 41 mg/100 g [4]. These values were comparable to the content of phenolic compounds in the analyzed market orange and green whey beverages, due to the similar amount of raw material used to manufacture the product.

The content of flavonoids in homemade and market whey beverages was not proportional to the amount of phenolic compounds. The highest amount of flavonoid compounds was identified in the homemade orange whey beverage (W1O), while the highest amount of polyphenols was determined in the homemade green whey beverage (W1G). The high content of flavonoids in the homemade orange whey beverage is determined by the addition of wild rose, which contains large amounts of catechin, belonging to the flavonoid group [14] as well as 25% of sea buckthorn, which is particularly rich in quercetin and its derivatives [15] and catechnia, kaempferol and isoramnetin, classified as flavones [16]. Much lower amounts of flavonoids in market whey beverages resulted from the much lower proportion of plant-based raw materials added. Flavonoids are considered to be one of the largest groups of secondary metabolites with strong antioxidant properties, however they are found only in products of plant origin (like polyphenols) [17].

In the study, the% of inhibition indicating the antioxidant capacity of homemade and market whey beverages, was for W1O, W1R, W1G, W2O, W2R, W2G: 88, 87, 89, 27, 18 and 32%, respectively. Abdul Alim et al. [18] examining the antioxidant activity of whey beverages with the DPPH test with the addition of 25% and 50% of black mulberry beverage, obtained the % of inhibition values at the level of 32% and 57%. The% of inhibition value of whey beverages under study was by 30% higher than in whey beverages with the addition of 50% black mulberry beverage. However the % of inhibition of market orange and green whey beverages was similar to the % of inhibition of whey beverages studied by Abdul Alim et al. [18]. Arranz et al. [19] investigated the antioxidant activity of whey beverages enriched with rosemary extract. The antioxidant activity of the tested beverage, measured by the ORAC test, was over 2000 µM TE/100 g of beverage. The values of the antioxidant activity of homemade whey beverages obtained in own research, measured with the ORAC test, were even several dozen times lower. The reason for this was, among others, the use of other raw materials to enrich beverages. In the studies of the authors cited, except from rosemary, beverages were enriched with vitamin B12, astaxanthin and epigallocatechin gallate, classified as antioxidants with strong anti-radical properties. In this study, fruit and vegetables were used as an additive enriching the beverages. Chen et al. [20] investigated the antioxidant properties of herbs and vegetables and showed that herbs were characterized by a much higher level of antioxidant activity. Arranz et al. [19] used rosemary extract as an additive to whey beverages, which contained a concentrated content of antioxidants. In own research there were used raw fruits and vegetables, which due to the higher water content, contained a lower concentration of antioxidants.

Among the studied fruit and vegetable mousses, the red mousses R2 and R3 had the highest content of phenolic compounds. Both mousses contain apple and beetroot rich in phenolic compounds [21]. In addition, R2 mousse contains pomegranate beverage and goji berries, and R3 mousse the 9% addition of cherries, considered a very good source of polyphenolic compounds [22]. Moreover, the R3 mousse had the highest content of compounds from the flavonoid group due to its varied composition. Nawirska-Olszańska et al. [23] report that pumpkin mousses enriched with 10% cherry addition were characterized by the content of phenolic compounds at the level of 35.31 mg/100 g. R3 mousse contained more than twice the content of phenolic compounds compared to pumpkin-cherry mousse due to a more diverse composition. In the studies by Čížková et al. [24] puree with the addition of strawberries contained 48.5 mg/100 g of phenolic compounds and with the addition of blueberries 77.0 mg/100 g. The content of phenolic compounds in puree with the addition of blueberries was at the same level as in the red mousses R2 and R3. The lower polyphenol content in strawberry mousse may resulted from the addition of sugar to the product. Nawirska-Olszańska et al. [23] conducted research on the content of phenolic compounds in pumpkin mousses with 20% apple addition. The content of phenolic compounds in this mousse was at the level of 38.90 mg/100 g. In our own research, there was analyzed the antioxidant activity of mousse with carrot, pumpkin and pear, where the content of phenolic compounds was 55.86 mg/100 g. The higher content of phenolic compounds in this study is explained by the more diverse composition of the product, including addition of carrot, which has stronger antioxidant properties, including total phenolic content, than pumpkin [25]. The “green” mousses were characterized by the content of phenolic compounds in the range of 60.60–69.80 mg/100 g. In each of these mousses, apart from apples and bananas, there is spinach—a vegetable considered to be particularly rich in polyphenolic compounds [26]. The green mousses were characterized by a variable content of phenolic compounds. G1 mousse contained over 10 mg/100 g more flavonoids than G2 and G3 mousses. This resulted from the very diverse composition of G1 mousse, which in addition to the base material (apples and spinach) contain rich in flavonoids: avocado, zucchini and green peas [27,28,29].

The content of phenolic compounds in the study was slightly comparable to the contents presented in the literature. These differences arise from the enrichment of some mousses with vitamin C. The producer does not declare what amounts of vitamin C are added to the product, and these values significantly affect the measurement of the content of phenolic compounds. The Folin-Ciocalteau reagent reacts, i.a, with ascorbic and dehydroascorbic acid (DHA) as well as reducing sugars, often giving overestimated results [30].

The lowest levels of antioxidant activity were determined in O3, G1 and G3 mousses. O3 and G3 mousses contain bananas and apples, which are considered to be fruits with low and medium ability to reduce free radicals [31]. Filipiak-Florkiewicz and Dereń [32] report that the differences in the antioxidant activity in mousses depend on the species of fruit and vegetables supplementing their composition. In the studies of these authors, the lowest antioxidant activity tested with the ABTS test was characteristic for apple mousse (1140 µM/100 g), and the highest apple and rosehip mousse (1420 µM/100 g). Rosehip fruit is an excellent source of antioxidants, especially vitamin C and phenolic compounds [33] and caused a significant increase in antioxidant activity in the studied mousses.

The content of individual lipophilic pigments (β-carotene, chlorophyll a, chlorophyll b) varied depending on the composition of the beverage or mousse. β-carotene is a dye belonging to the group of carotenoids, the carotenes class, considered to be one of the most important dyes in fruits and vegetables. The richest plant source of β-carotene is carrot and green leafy vegetables—parsley, kale and spinach [1]. The market orange whey beverage (W1O) contained the highest content of β-carotene due to the addition of carrot puree. The homemade green whey beverage (W1G) also accumulated β-carotene due to the addition of parsley. These vegetables, apart from enriching the taste of beverages, significantly increased their nutritional value. Market orange whey beverage (W2O) contained mango and passion fruit puree, which are also considered a good source of β-carotene [34], but their addition was only 8%, therefore the β-carotene content was not significantly high. Orange mousses contained the highest amounts of β-carotene, due to the addition of carrots, pumpkins and concentrated orange beverage. The content of β-carotene in orange mousses was strictly determined by their composition. There were tendencies that the more varied the composition of the mousse, the higher β-carotene content. Orange mousse O2 was characterized by the highest content of this compound—in addition to carrots rich in β-carotene—it also contained pumpkin, peach and apricot, which are considered to be an equally good source of this carotenoid [35] Among green purees, G1 mousse contained the highest amount of β-carotene, due to the relatively high proportion of spinach in its composition. Red mousses, due to the lack of raw materials rich in carotenoids, contained very small amounts of β-carotene. Chlorophylls belong to the group of green dyes and are the main dye of green leafy vegetables. There are two types of chlorophyll in the chloroplasts of higher plants—chlorophyll a (blue-green) and chlorophyll b (yellow-green)—which differ in their chemical structure. In whey beverages, the highest amounts of chlorophyll a and b were contained in the whey beverage Z, which results from a significant amount of parsley in it. Parsley is a leafy vegetable particularly rich in bioactive compounds—polyphenols, vitamins C and A, and chlorophylls [36]. Compared to the homemade green whey beverage (W1G), the market green whey beverage (W2G) contained a very low chlorophyll content, which was influenced by the composition of the beverage, which was low in vegetables and fruits rich in chlorophylls. The content of chlorophyll in homemade orange and red whey beverages was negligible, which is related to a small amount of raw materials rich in chlorophyll in their composition. Green mousses were the richest source of chlorophylls among all mousses. These mousses in addition to the base ingredient—apple, contained spinach, avocado, zucchini, green peas or kiwi. This raw materials, except from diversified the taste of mousses, enriched them with chlorophyll dyes. G1 mousse contained the highest amount of chlorophyll due to the 10% addition of spinach and 5% of avocado in its composition. Mitić et al. [37] examining green leafy vegetables, showed that spinach accumulated the highest amounts of chlorophyll a and b.

## 4. Materials and Methods

### 4.1. Chemical and Reagents

2,2′-Azobis(2-amidopropane) hydrochloride (AAPH), 2,2-diphenyl-1-picrylhydrazyl (DPPH) and 6-hydrozy-2,5,7,8-tetramethylchroman-2-carboxylic acid (Trolox) were purchased from Sigma Chemical Co. (St. Louis, MO, USA). Sodium fluorescein, phosphate-buffered saline (PBS), sodium thiosulfate and sodium hydroxide were obtained from Chempur (Piekary Śląskie, Poland). Hexane, acetone, ethanol, methanol, toluene, isopropanol, Folin-Ciocalteu reagent, gallic acid, catechin, sodium nitrite and aluminium chloride were purchased from Sigma Aldrich (St. Louis, MO, USA).

### 4.2. Research Material

The acid whey was obtained during the traditional production of semi-skimmed curd from cow’s milk pasteurized at 83 °C for 15 min. Whey was used to make homemade fruit and vegetable whey beverages. Three variants of this type of beverages were obtained, containing approx. 50% of acid whey and 50% of other ingredients (carrot puree, roseship puree, sea buckthorn jam in orange beverage; pear puree and concentrated cherry juice in red beverage; parsley and banana puree in green beverage). The beverages were obtained by thoroughly mixing the whey with the remaining components using blender (Braun MQ7035, Kronberg im Taunus, Germany). The analysis also covered market whey beverages from Piątnica and fruit and vegetable mousses of the following brands: Bobovita, Bobofrut, Gerber, Hortex, Dawtona, Freche Freunde. The products were purchased on the Olsztyn market in one of the supermarkets. The composition of the products used for the analyzes is presented in Table 3. The abbreviations used in the experiment are: W1O, W1R and W1G for homemade fruit and vegetable whey beverages; W2O, W2R and W2G for the market fruit and vegetable whey beverages; O1, O2, O3; R1, R2, R3; G1, G2, G3 for orange, red and green fruit and vegetable mousses, respectively.

For the analysis, 5 g of the research materials was diluted five times with distilled water and then centrifuged in an Eppendorf type centrifuge (Eppendorf AG, Hamburg, Germany) (4000 rpm/10 min). Prepared extracts were used to determine the antioxidant activity by DPPH and ORAC tests and total phenolic contents (TPC) and total flavonoid content (TFC). For the determination of lipophilic pigments (β-carotene and chlorophylls) the acetone-hexane extracts were prepared.

### 4.3. Determination of Antioxidant Activity by DPPH Test

The determination of the antioxidant activity by the DPPH method was carried out according to the procedure developed by Yang et al. [38]. A solution of the DPPH radical (2,2-diphenyl-1-picrylhydrazyl) was prepared by dissolving 27.8 mg of DPPH in 200 mL of 80% methanol. In order to perform the spectrophotometric test, 100 μL of the examined extract and 900 μL of DPPH solution were mixed. The mixture was left for 16 min at room temperature. The absorbance at 515 nm was measured with a FLUOstar OMEGA BGM LAB Tech spectrophotometer (Ortenberg, Germany) against the reagent test. The reagent sample contained distilled water instead of the tested extract. The antioxidant activity of the tested samples was expressed in µM of Trolox/100 g of product, against the standard curve prepared for the Trolox solution.

### 4.4. Determination of Antioxidant Activity by ORAC Test

The determination of the antioxidant activity by the ORAC method was carried out as described in Huang et al. [39] article, with some modifications. To 25 µL of the extract 150 µL of a 10nM solution of fluorescein in buffered salt solution (PBS) was added. All was incubated for 15 min at 37 °C. After incubation, 25 µL of 240 nM AAPH in PBS was added to start the reaction. Fluorescence (excitation at wavelength 485 nm, emission at wavelength 520 nm) was measured every 90 s for 6 h. To process the results, the absorbance of the reagent sample was also measured. The reagent sample contained distilled water instead of the tested extract. The antioxidant activity of analyzed samples was expressed in µM of Trolox/100 g, against the standard curve prepared for the Trolox solution.

### 4.5. Determination of Total Phenolic Content (TPC)

The modified Ribereau-Gayon [40] method was used to determine the total phenolic compounds content. In order to perform the determination, 0.5 mL of the analyzed extract was measured into a 10 mL volumetric flask. 0.5 mL of Folin-Ciocalteu reagent (diluted 1:2 with distilled water) was added and mixed. Then 3 mL of a 14% sodium carbonate solution was added and mixed. The mixture was made up to 10 mL with distilled water and mixed again. The prepared solutions were left for 60 min in the darkened place. After this time, the absorbance of the solutions was measured at 720 nm with the FLUOstar OMEGA BGM LAB Tech spectrophotometer against the reagent sample. The reagent sample contained distilled water instead of the analyzed extract. The total phenolic compounds content was expressed in mg of catechin/100 g of product, against the standard curve prepared for the catechin solution.

### 4.6. Determination of Total Flavonoid Content (TFC)

To determine the total flavonoid content the method developed by Herald, Gadgi and Tilley [41] and Yafang, Gan and Jinsong [42] was used. To a 15 mL polypropylene test tube containing 4 mL of deionized water, 1 mL of the analyzed extract and 0.3 mL of a 5% NaNO2 solution were added. The whole was mixed thoroughly and left for 5 min for reaction. Then, 0.6 mL of 10% AlCl_3_ 6H_2_O solution was added to the mixture and allowed to stand for 6 min. After the reaction, 2 mL of 1 M NaOH solution and 2 mL of deionized water were added. The mixture was centrifuged in an Eppdendorf centrifuge type 5810R (Eppendorf AG, Hamburg, Germany) at 4000 rpm for 10 min. The absorbance of the mixture was measured at 415 nm using a UV/Vis UV2 spectrophotometer (Unicam, Ortenberg, Germany) against the reagent sample. The reagent sample contained distilled water instead of the analyzed extract. The content of flavonoids was expressed in mg of catechin/100 g of product, against the standard curve prepared for the catechin solution.

### 4.7. Determination of Lipophilic Pigments

The method described by Marx, Schieber and Carle [43] was used for determine the content of lipophilic pigments, including carotenoids and chlorophylls. Beverages and mousses sample (5 g) was extracted three times in an amber glass separatory funnel with a 30 mL mixture of acetone and hexane (1:1, *v*/*v*). The emulsion formed was removed by adding 50 mL sodium chloride solution (10%, *w*/*v*). After separation, the hexane layer was washed three times with water (50 mL) to remove acetone. The extract was dried with sodium sulfate (2 g). The separated hexane phase was evaporated to dryness under a nitrogen stream at 40 °C. The absorbance of the centrifuged mixture was measured and the absorption maxima were read at 453, 645 and 663 nm using a Unicam UV/Vis UV2 spectrophotometer against the reagent sample. The reagent sample contained acetone and hexane (1:1, *v*/*v*) instead of the analyzed extract. β carotene, chlorophyll a and chlorophyll b content was calculated from the following Equations (1)–(3) [44]:

β-Carotene (mg/100 g) = 0.216 A_663_ − 1.22 A_645_ − 0.304 A_505_ + 0.452 A_453_ (1)

Chlorophyll a (mg/100 g) = 0.999 A_663_ − 0.0989 A_645_ (2)

Chlorophyll b (mg/100 g) = −0.328 A_663_ + 1.77 A_645_ (3)

### 4.8. Statistical Analysis

The values were expressed as mean ± standard deviation (SD). The results were subjected to a one-way analysis of variation (ANOVA) using Duncan’s test. A linear Pearson’s correlation coefficients were calculated to show relationships between bioactive compounds and antioxidant capacity, and *p* < 0.05 was considered significant. Principal Component Analysis (PCA) was also carried out to show differences between samples. The statistical analysis was performed using Statistica 13.1 (Statsoft Inc., Tulsa, OH, USA).

## 5. Conclusions

The antioxidant activity and the content of selected antioxidants in analyzed fruit and vegetable products (whey beverages, mousses) were determined by the composition and percentage share of individual plant raw materials. Homemade whey beverages, due to the higher addition of fruit and vegetable components (up to 50%), were characterized by significantly higher antioxidant activity and content of phenolic compounds, flavonoids and lipophilic pigments. The addition of whey in fruit and vegetable beverages highly contributes to the enhancing of their nutritional value. Whey is a rich source of minerals, among others calcium, phosphorus, sodium and potassium. Taking into account the results of the obtained research, it is worth using a higher addition of fruit and vegetable components in dairy products available on the market. It is a valuable indication for food producers to enrich dairy products with higher amounts of fruit and vegetable components to increase their health-promoting properties. Enrichment of dairy products with antioxidant compounds, including polyphenols, carotenoids and chlorophylls, significantly increases their antioxidant potential and improves taste.

## Figures and Tables

**Table 1:** Antioxidant properties of fruit and vegetable whey beverages and fruit and vegetable mousses.

		TPC [mg/100 g]	TFC [mg/100 g]	Antioxidant Activity Assays [µM TE/100 g]		Lipophilic Pigments [mg/100 g]		
				DPPH	ORAC	β-Carotene	Chlorophyll a	Chlorophyll b
Beverages	W1O	428.05 ± 8.87 ^B^	63.06 ± 2.81 ^A^	78.75 ± 3.22 ^A^	71.58 ± 2.29 ^A^	4.36 ± 0.66 ^A^	0.02 ± 0.00 ^C^	0.04 ± 0.01 ^C^
	W1R	247.61 ± 2.63 ^C^	35.34 ± 0.41 ^B^	78.11 ± 0.42 ^A^	73.55 ± 1.19 ^A^	ND	0.09 ± 0.01 ^B^	0.14 ± 0.00 ^B^
	W1G	541.95 ± 0.74 ^A^	37.10 ± 0.25 ^B^	79.32 ± 1.95 ^A^	70.76 ± 3.21 ^A^	0.77 ± 0.11 ^B^	3.00 ± 0.23 ^A^	1.31 ± 0.01 ^A^
	W2O	49.28 ± 1.30 ^E^	12.40 ± 0.00 ^C^	11.03 ± 0.98 ^B^	51.46 ± 1.70 ^C^	0.21 ± 0.07 ^C^	0.01 ± 0.00 ^C^	0.02 ± 0.01 ^C^
	W2R	68.96 ± 0.88 ^D^	12.52 ± 0.00 ^C^	7.35 ± 1.07 ^C^	53.1 ± 0.54 ^C^	ND	0.09 ± 0.02 ^B^	0.14 ± 0.02 ^B^
	W2G	46.18 ± 4.17 ^E^	14.96 ± 0.25 ^C^	13.09 ± 5.11 ^B^	63.02 ± 0.19 ^B^	0.02 ± 0.00 ^D^	0.02 ± 0.00 ^C^	0.05 ± 0.02 ^C^
Mousses	O1	53.18 ± 4.05 ^c^	15.70 ± 0.58 ^b^	41.16 ± 1.71 ^c^	45.56 ± 0.16 ^b^	1.25 ± 0.26 ^b^	0.04 ± 0.01 ^e^	0.04 ± 0.01 ^d^
	O2	55.86 ± 0.58 ^c^	13.13 ± 0.08 ^c^	37.12 ± 1.10 ^c^	47.21 6.53 ^b^	2.24 ± 0.55 ^a^	0.07 ± 0.02 ^e^	0.11 ± 0.06 ^c^
	O3	56.04 ± 2.16 ^c^	18.91 ± 0.17 ^a^	27.05 ± 2.66 ^d^	57.08 ± 2.21 ^a^	1.10 ± 0.02 ^f^	0.07 ± 0.02 ^e^	0.07 ± 0.03 ^d^
	R1	56.82 ± 1.06 ^c^	12.29 ± 0.33 ^c^	36.19 ± 2.10 ^c^	49.09 ± 6.25 ^b^	0.08 ± 0.03 ^f^	0.15 ± 0.07 ^d^	0.10 ± 0.01 ^c^
	R2	71.22 ± 3.39 ^b^	15.38 ± 0.24 ^b^	57.54 ± 2.17 ^a^	32.17 ± 5.49 ^c^	0.08 ± 0.01 ^f^	0.05 ± 0.01 ^e^	0.04 ± 0.01 ^d^
	R3	76.41 ± 4.00 ^a^	10.75 ± 0.74 ^d^	53.29 ± 4.43 ^b^	47.15 ± 6.62 ^b^	0.13 ± 0.00 ^f^	0.02 ± 0.00 ^e^	ND
	G1	60.60 ± 2.02 ^c^	19.76 ± 0.83 ^a^	26.39 ± 4.21 ^d^	41.97 ± 0.16 ^b^	0.76 ± 0.16 ^c^	1.42 ± 0.22 ^a^	0.19 ± 0.00 ^b^
	G2	69.80 ± 2.78 ^b^	19.90 ± 0.41 ^a^	49.03 ± 4.83 ^b^	46.09 ± 2.97 ^b^	0.23 ± 0.08 ^e^	0.54 ± 0.12 ^c^	0.32 ± 0.16 ^a^
	G3	64.27 ± 1.11 ^b^	19.26 ± 0.83 ^a^	25.13 ± 1.13 ^d^	21.63 ± 2.26 ^d^	0.31 ± 0.06 ^d^	1.10 ± 0.11 ^b^	0.13 ± 0.01 ^c^

Explanation: W1—homemade fruit and vegetable whey beverage; W2—market fruit and vegetable whey beverage; O—orange; R—red; G—green; ND—non detected. Means in the column with different letters (ABC/abc) are significantly different (*p* < 0.05).

**Table 2:** Correlation coefficients (r) calculated for the relationships between (r marked by * are statistically significant at *p* < 0.05).

Correlation	DPPH	ORAC	TPC	TFC	β-Carotene	Total Chlorophylls Content
DPPH	1	0.43	0.77 *	0.61 *	0.40	0.32
ORAC	0.43	1	0.68 *	−0.03	0.28	0.14
TPC	0.77 *	0.68*	1	0.34	0.48	0.61 *
TFC	0.61 *	−0.03	0.34	1	0.53 *	0.05
β-carotene	0.40	0.28	0.48	0.53 *	1	−0.04
Total chlorophyll content	0.46	0.31	0.60 *	0.53 *	0.98 *	1

**Table 3:** Percentage composition of the fruit and vegetable whey beverages and fruit and vegetable mousses.

Type of Product	Symbol	Percentage of Individual Components
Homemade whey beverages	W1O	Acid whey (50%), carrot puree (25%), rosehip puree (12.5%), sea buckthorn jam (12.5%)
	W1R	Acid whey (50%), pear puree (37.5%), concentrated cherry juice (12.5%)
	W1G	Acid whey (50%), parsley (25%), banana puree (25%)
Market whey beverages	W2O	Acid whey, fruits 8% (mango puree 5%, passion fruit puree 3%), sugar, gluten-free oats 0.05%, natural aromas
	W2R	Acid whey, fruits 8% (raspberry puree 5.2%, pomegranate juice 2.8%), sugar, natural aromas, black carrot juice concentrate, ginseng root extract 0.02%
	W2G	Acid whey, fruits 6.5% (banana juice 3.5%, banana 1.5%, gooseberry 1.5%), sugar, fiber, natural aroma, carrot concentrate
Mousses	O1	Apple (62%), carrot (35%), apple juice concentrate (5%), vitamin C
	O2	Carrot (30%), pear (26%), pumpkin (14%), peach (12%), apricot (12%), parsnip (6%), vitamin C, lemon juice concentrate
	O3	Banana (42%), pear (38%), apple (15.4%), concentrated juices (orange 2.5%, pear 1.8%, lemon), vitamin C
	R1	Apple (90%), beetrot (10%), vitamin C
	R2	Apple (54%), banana (32%), beetrot (8%), concentrated pomegranate juice (5%), goji berries (1%), vitamin C
	R3	Apple (58%), banana (25%), cherry (9%), beetrot (8%), vitamin C
	G1	Apple (62%), spinach (10%), avocado (8%), zucchini (7.5%), green peas (7.5%), banana (5%), vitamin C, lemon juice concentrate
	G2	Apple (65%), zucchini (15%), banana (10%), spinach (5%), kiwi (5%), vitamin C
	G3	Apple (53%), banana (22%), spinach (17%), cucumber juice (8%), lemon juice (<0.5%)

Explanation: W1—homemade fruit and vegetable whey beverage; W2—market fruit and vegetable whey beverage; O—orange; R—red; G—green.

## Data Availability

The data presented in this study are available on reasonable reguest from the corresponding author.

## Sample Availability

Samples of the compounds are not available from the authors.
